# K^+^ and Cl^−^ channels/transporters independently fine-tune photosynthesis in plants

**DOI:** 10.1038/s41598-019-44972-z

**Published:** 2019-06-14

**Authors:** Emilija Dukic, Andrei Herdean, Otilia Cheregi, Anurag Sharma, Hugues Nziengui, Dominika Dmitruk, Katalin Solymosi, Mathias Pribil, Cornelia Spetea

**Affiliations:** 1 Department of Biological and Environmental Sciences, University of Gothenburg, Box 461, Gothenburg, 40530 Sweden; 2 Copenhagen Plant Science Centre, Department of Plant and Environmental Sciences, University of Copenhagen, Thorvaldsensvej 40, DK-1871 Frederiksberg C, Copenhagen, Denmark; 3 Department of Botany, Warsaw University of Life Sciences, Nowoursynowska 166, Warsaw, 02-787 Poland; 4 Department of Plant Anatomy, ELTE - Eötvös Loránd University, Pázmány P. s. 1/C, Budapest, H-1117 Hungary

**Keywords:** Chloride channels, Chloroplasts, Light responses, Non-photochemical quenching, Photosystem II

## Abstract

In variable light environments, plants adjust light use in photosynthetic electron transport and photoprotective dissipation in the thylakoid membrane. In this respect, roles of the K^+^/H^+^ antiporter KEA3, the Cl^−^ channel/transporter CLCe and the voltage-dependent Cl^−^ channel VCCN1 have been unraveled in *Arabidopsis thaliana*. Here we report that they independently adjust photosynthesis on the basis of analyses using single and higher order loss-of-function mutants. In short experiments of photosynthetic response on transition from dark to low light, we reveal a sequential functioning of VCCN1 and CLCe in the activation of photoprotection and of KEA3 in its downregulation to a low steady state while adjusting the electron transport. On transition from low to high light, VCCN1 accelerates the activation of photoprotection, whereas KEA3 slows it down on transition from high to low light. Based on parallel electrochromic band shift measurements, the mechanism behind is that VCCN1 builds up a pH gradient across the thylakoid membrane, whereas KEA3 dissipates this gradient, which affects photoprotection. CLCe regulates photosynthesis by a pH-independent mechanism likely involving Cl^−^ homeostasis. Nevertheless, all genotypes grow well in alternating high and low light. Taken together, the three studied ion channels/transporters function independently in adjusting photosynthesis to the light environment.

## Introduction

Plants harvest sunlight and convert the captured energy into biochemical products. For the energy conversion to take place, several processes occur in a timely manner in what is generically called photosynthetic light-dependent reactions. Sunlight is not only the energy source for photosynthesis but also an important limiting factor due to its fluctuations in intensity^1^. Rapid and abrupt changes in light conditions affect electron transport through photosystem II (PSII), cytochrome b_6_f and photosystem I (PSI) complexes in the thylakoid membrane^2^. To overcome the constraints imposed by sunlight, energy capture and dissipation of excess light energy are tightly regulated during photosynthesis. In order to avoid damage to photosystems, excess energy is quenched by dissipation as heat in a mostly pH-dependent process known as qE-type of non-photochemical quenching (NPQ)^3^. NPQ undergoes activation and downregulation in response to changes in light intensity, and is controlled by the pH in the thylakoid lumen, the pH sensor protein PsbS and the carotenoid zeaxanthin^4^. The rates of NPQ activation and in particular of its downregulation are rather slow (order of minutes) as compared to natural light fluctuations (order of seconds), and are likely limited by the slow kinetics of pH changes in the thylakoid lumen^5^. Several *Arabidopsis thaliana npq* mutants have been discovered, but they grew similarly to wild-type plants under constant laboratory light conditions^6,7^. Nevertheless, experiments in field conditions with the same mutants indicated that NPQ confers increased plant tolerance to fluctuating light^1^. Moreover, tobacco mutants engineered to accelerate NPQ response to fluctuating light showed improved photosynthesis and biomass production^8^.

There is increasing evidence that NPQ is also regulated by ion channels/transporters able to modulate the kinetics of pH changes in the lumen^9,10^. In the thylakoid membrane of *Arabidopsis*, the two-pore K^+^ channel TPK3 is essential for activation of NPQ^11^, whereas the K^+^/H^+^ antiporter KEA3 accelerates its downregulation and stimulates the electron transport^12^. The mechanism behind the action of both proteins is thought to be an altered K^+^:H^+^ stoichiometry in the lumen, which in turn changes the lumenal pH. Armbruster and colleagues^13^ provided experimental evidence for the activation of KEA3 by its C-terminus upon sudden shifts to low light intensities. The Cl^−^ channel/transporter CLCe regulates the electron transport^14,15^, whereas the voltage-dependent Cl^−^ channel VCCN1 accelerates the activation of NPQ in conditions of increasing light intensities^16,17^. To explain these observations, Cl^−^ influx is thought to electrically balance the H^+^ uptake into the thylakoid lumen, which in turn favors additional H^+^ uptake and hence lumen acidification. In addition, ion channels/transporters are important for thylakoid ultrastructure^11,14,16–18^, that may also influence photosynthetic energy conversion^19^.

Photosynthetic electron transport is coupled with H^+^ uptake into the lumen, generating an electrochemical gradient (or proton motive force, PMF) across the thylakoid membrane. The PMF is composed of an electric field (∆Ψ) and a pH gradient (∆pH), both driving the synthesis of ATP^20^. They have also distinct roles since a high ∆pH activates NPQ and inhibits electron transport^4^, whereas a high ∆Ψ enhances the rate of charge recombination and PSII photodamage^21^. Loss-of-function of each of the identified thylakoid-located channels/transporters altered the PMF partitioning into ∆pH and ∆Ψ which influenced the balance between light use in electron transport and photoprotective heat dissipation^11,12,14,16–18^. Moreover, since the KEA3 loss-of-function maintains high NPQ only in low light conditions^12^, whereas the lack of VCCN1 and CLCe diminishes NPQ and lowers electron transport, respectively, at all light intensities^14,17^, distinct mechanisms in the action of the three proteins can be hypothesized. In this study, we characterized single, double and triple loss-of-function mutants in *Arabidopsis* and show that KEA3, CLCe and VCCN1 function independently to fine-tune photosynthesis in response to changes in the light environment.

## Results

### Growth and photosynthesis phenotype under standard light conditions

To better understand the role of CLCe, KEA3 and VCCN1 and their possible interdependency in the regulation of PMF and photosynthesis, we compared the phenotypes of *clce-2, kea3-1* and *vccn1-1* single loss-of-function mutant lines^14,17,18^, hereafter named *clce, kea3* and *vccn1*, with those of newly generated *clcekea3, clcevccn1, kea3vccn1* double and *clcekea3vccn1* triple mutant lines.

**Figure 1:**
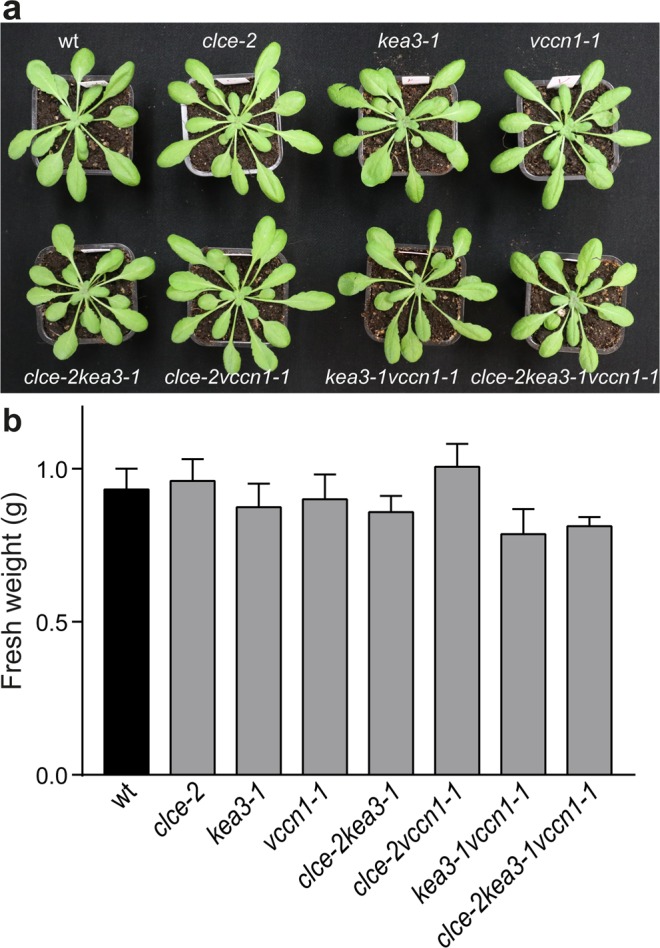
Growth phenotype of 5-week-old plants watered with deionized water. (**a)** Representative photos of mutants cultivated using a 8 h/16 h light/dark cycle and a light intensity of 120 μmol photons m^−2^ s^−1^ show no difference in growth with respect to wild-type (wt) plants. (**b**) Shoot biomass of 5-week-old plants including those shown in (a) was determined as fresh weight. Data are the means ± SEM (n = 12 plants). There was no significant difference between wt and mutants (one-way ANOVA test, P > 0.05). The experiment was repeated twice with similar results.

All mutant lines grew similarly to wild-type (wt) plants under standard light conditions (120 µmol photons m^−2^ s^−1^) and displayed similar shoot fresh weight at 5 weeks (Fig. 1a,b) and 8 weeks (Supplementary Fig. 1a,b). The chlorophyll (Chl) content and Chl *a*/*b* ratio were also similar to wt at both ages (Table 1 and Supplementary Table 1).

**Table 1:** Chlorophyll content and fluorescence parameters of 5-week-old plants watered with deionized water.

Parameter	wt	*clce-2*	*kea3-1*	*vccn1-1*	*clce-2kea3-1*	*clce-2vccn1-1*	*kea3-1vccn1-1*	*clce-2kea3-1vccn1-1*
μg Chl cm^−2^	11.71 ± 0.43	11.67 ± 0.95	11.89 ± 0.63	13.40 ± 0.10	11.80 ± 0.43	12.52 ± 0.63	12.67 ± 0.63	13.22 ± 0.51
Chl *a/b*	2.76 ± 0.03	2.77 ± 0.02	2.82 ± 0.04	2.78 ± 0.02	2.81 ± 0.02	2.78 ± 0.03	2.80 ± 0.04	2.73 ± 0.03
F_v_/F_m_	0.819 ± 0.008	0.802 ± 0.001*	0.813 ± 0.000	0.813 ± 0.000	0.800 ± 0.001*	0.799 ± 0.001*	0.813 ± 0.000	0.797 ± 0.001*
PI_ABS_	1.99 ± 0.03	1.83 ± 0.04*	2.05 ± 0.03	2.04 ± 0.02	1.78 ± 0.04*	1.81 ± 0.04*	1.99 ± 0.04	1.74 ± 0.03*
t_Fm_ (ms)	225 ± 30	566 ± 173*	259 ± 30	261 ± 30	438 ± 165*	503 ± 190*	242 ± 34	592 ± 164*
Y(II)	0.642 ± 0.004	0.613 ± 0.006*	0.637 ± 0.005	0.626 ± 0.007	0.615 ± 0.005*	0.607 ± 0.007*	0.628 ± 0.005	0.602 ± 0.005*

Plants were grown as described in Fig. 1. Leaf Chl content and Chl *a/b* ratio were determined spectrophotometrically from leaf discs of 16-h dark-adapted wild-type plants (wt) and mutants following extraction in ethanol. The maximal quantum yield of PSII photochemistry (F_v_/F_m_), the photosynthetic performance index (PI_ABS_) and the time to reach F_m_ (t_Fm_) were estimated from fast kinetics of Chl fluorescence in 30 min dark-adapted plants. The PSII quantum yield (Y(II)) was determined under steady-state growth light conditions (4 h of illumination at 120 μmol photons m^−2^ s^−1^). Data are means ± SEM from 3 experiments (n = 10 plants per experiment). Asterisks indicate statistically significant difference in the studied parameters between wt and mutants (ANOVA, P < 0.05).

**Figure 2:**
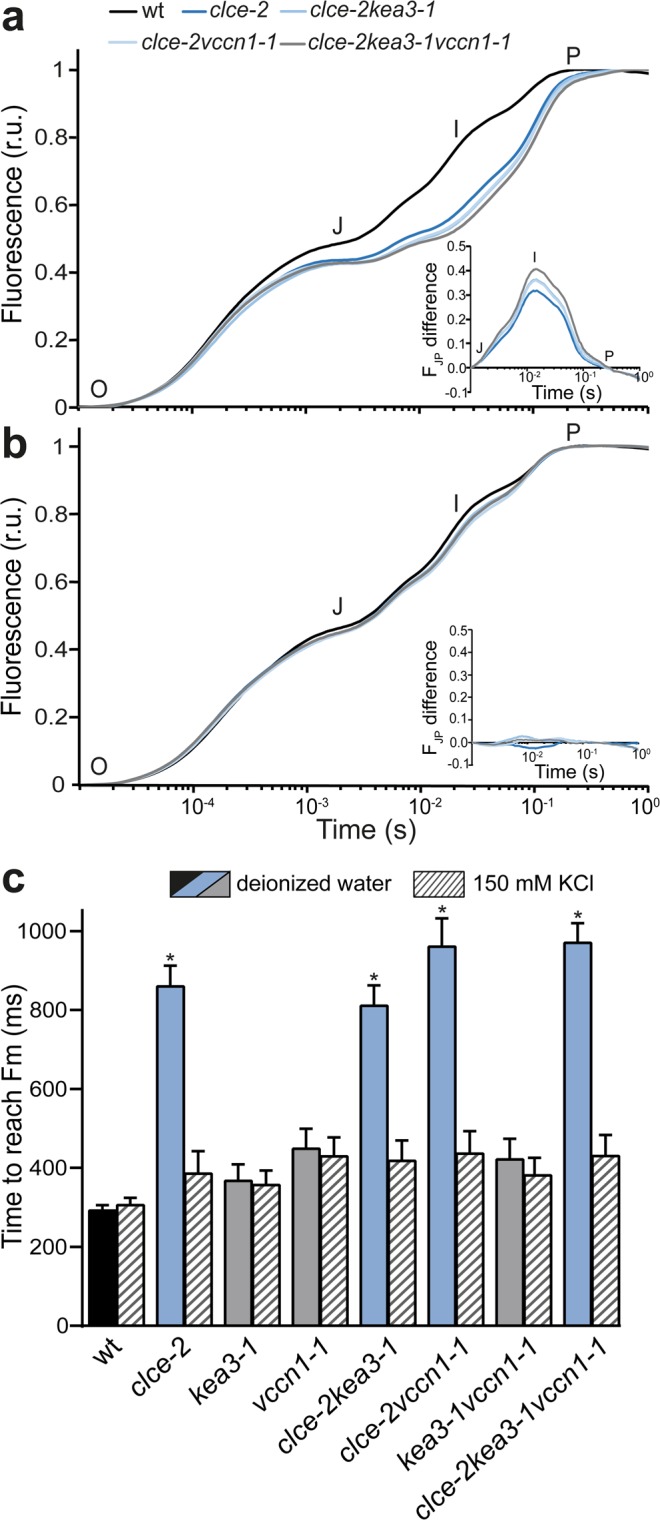
Fast chlorophyll *a* fluorescence *OJIP* transients. Wild-type plants (wt) and mutants were grown for 5 weeks and watered with deionized water. *OJIP* transients were recorded on 15 min dark-adapted leaves which were pre-incubated for 30 min in deionized water (**a**) or in deionized water supplemented with 150 mM KCl (**b**) in the growth light. The transients were double normalized to the levels of *O* and *P* steps corresponding to F_0_ and F_m_. *Insets* show the curve difference of double normalized data at *J* and *P* steps between wt and each of the *clce* mutant lines. (**c**) The parameter corresponding to the time necessary to reach maximal fluorescence intensity F_m_ was calculated from the *OJIP* transients of (**a,b)**. Data are the means ± SEM (n = 8–10 plants). Asterisks on blue bars denote a statistically significant difference between wt and *clce* mutant lines in the same treatment (one-way ANOVA test, P < 0.05). The experiment was repeated twice with similar results.

To determine the maximal photosynthetic efficiency in terms of electron transport, we recorded fast Chl fluorescence (*OJIP*) kinetics on intact leaves of dark-adapted plants. At 5 weeks, the curve in wt had a polyphasic shape, while lower fluorescence levels at the *J* and *I* steps, previously reported in *clce*^14,15^, were also reproduced in this study and retained in *clce* double and triple mutant lines (Fig. 2a). The curves were double normalized to *J* and *P* and the curve difference between wt and *clce* lines was plotted. The resulting peak corresponds to the *I* step (Fig. 2a *inset*), associated with electron transport at PSI^22^. Double normalization to *O* and *J* reflecting PSII antenna size and activity resulted in much smaller differences between wt and the *clce* lines as compared to those in the *I* peak (Supplementary Fig. 2). In addition, the maximal quantum yield of PSII (F_v_/F_m_), the photosynthetic performance index (PI_ABS_, an overall indicator of efficiency of photosynthesis), and the time to reach maximal fluorescence intensity (t_Fm_) were similar to wt in all mutants with the exception of *clce* lines (Table 1). F_v_/F_m_ and PI_ABS_ in *clce* were slightly but significantly reduced, whereas t_Fm_ was over 2-fold higher, indicating a slower rate to close all PSII centers. This phenotype was preserved in *clcekea3* and *clcevccn1* double and *clcekea3vccn1* triple lines as well. We also recorded oxidation-reduction kinetics of P700 (PSI primary donor) and found a more pronounced reduction of P700^+^ in dark-incubated *clce* single and *clcekea3vccn1* triple mutant lines than in wt (Supplementary Fig. 3), in line with previous findings^14^_._ This suggests an accelerated electron transfer between PSII and PSI and could explain the lower fluorescence levels at the *J* and *I* steps (Fig. 2a) and higher values for t_Fm_ in *clce* lines (Table 1). The observed differences are typical for the dark state since *clce* plants adapted to standard (growth) light were indistinguishable from wt in P700^+^ reduction kinetics (Supplementary Fig. 3) as well as in the light response curves of the PSI quantum yield (Y(I), Supplementary Fig. 4).

We also recorded Chl fluorescence in plants under steady-state standard light conditions and again found that the *clce* lines differed from the other genotypes after 5 weeks of growth. The PSII quantum yield (Y(II)) as measure of the effective electron transport of PSII was slightly but significantly reduced under these conditions (Table 1).

The photosynthetic phenotype of *clce* was observed in plants grown for 5 weeks in standard light and watered with deionized water (Table 1). Remarkably, this phenotype disappeared in older *clce* plants (8 weeks) as based on the non-significant differences from the wt for the Chl fluorescence parameters (Supplementary Table 1), in accordance with previous observations^10^. We further found that when tap water was used (1–5 mM Cl^−^), 6-week-old *clce* lines showed Chl fluorescence and t_Fm_ values indistinguishable from wt (Supplementary Fig. 5). This may be due to increased accumulation of Cl^−^ in leaves with age and watering conditions, which compensate for the disturbed Cl^−^ distribution in *clce* lines. In support of this hypothesis, detached leaves of 5-week-old *clce* lines that were pre-incubated in deionized water supplemented with Cl^−^ (150 mM) displayed normal Chl fluorescence and t_Fm_ similar to wt (Fig. 2b,c), in accordance with previous findings^14^.

To further strengthen our hypothesis about a disturbed Cl^−^ homeostasis in *clce*, in a separate set of experiments we have grown plants with different Cl^−^ supply in the watering solution until the age of 6 and 8 weeks. The plants supplied with 0.5 mM Cl^−^ preserved the fluorescence phenotype observed with deionized water at 6 but not at 8 weeks, whereas plants supplied with 5 mM Cl^−^ resembled wt at both ages (Supplementary Figs. 6, 7). Since we obtained consistent fluorescence phenotypes with reproducible data when the *clce* plants were watered with deionized water at the age of 5–6 weeks, we used this watering procedure for all genotypes throughout the experiments in this study. Taken together, our observations from experiments on the dark- and light-adapted plants indicate that all lines grow well and perform wt-like photosynthesis under standard light conditions with the exception of *clce*. The *clce* lines experience an altered Cl^−^ homeostasis which impacts photosynthetic electron transport, without, however, affecting the capacity to produce biomass.

### Regulation of photosynthesis on transition from dark to light

**Figure 3:**
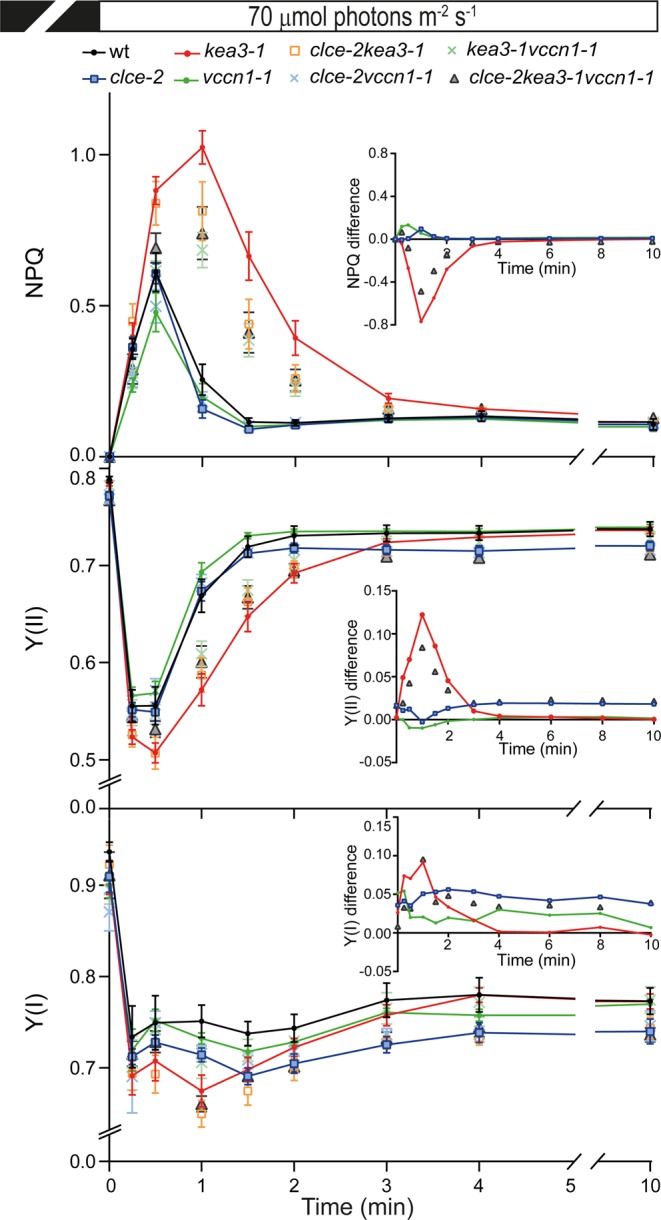
Dynamics of photosynthesis on transition from dark to low light. Wild-type plants (wt) and mutants were grown for 5 weeks and watered with deionized water. Kinetics for induction of non-photochemical quenching (NPQ) as a measure of photoprotection were recorded during 10 min of illumination at 70 µmol photons m^−2^ s^−1^. Photosystem II and I quantum yields (Y(II) and Y(I), respectively), as measures of the electron transport, were calculated from the same experiment as NPQ. *Insets* show the curve difference of wt minus each mutant. For clarity, only data for single and triple mutants are shown. For NPQ difference plots of double mutants, see Supplementary Fig. 8. Data are the means ± SEM (n = 6–8 plants). The experiment was repeated twice with similar results.

Slow kinetics of Chl fluorescence induction were recorded to determine the role of CLCe, KEA3 and VCCN1 in the regulation of photosynthesis. Upon transition from dark to low light, NPQ transiently decreased in *vccn1* and *clce*, whereas in *kea3* it increased 2-fold and relaxed more slowly before reaching a steady state similar to wt (Fig. 3). Double and triple mutant lines displayed intermediate NPQ patterns, indicating additive effects of the mutations. To visualize the contribution of each individual channel/transporter to the kinetics of NPQ, we calculated the difference of wt minus mutant^23^. VCCN1 accelerated NPQ activation with a maximum at 0.5 min followed by CLCe with a maximum at 1 min of illumination. KEA3 relaxed most of the NPQ during 3 min of illumination. During the overlapping periods, NPQ became the sum of individual contributions, as evidenced by the intermediate NPQ difference in the double and triple mutant lines (Fig. 3 *inset*, Supplementary Fig. 8).

Photosynthetic electron transport measured as PSII and PSI quantum yield (Y(II) and Y(I), respectively) in the same experiment, was reduced in *kea3* during the 3 min corresponding to NPQ induction and relaxation, and was similar to wt during the steady state (Fig. 3). The *clce* displayed a reduced Y(I) throughout the illumination and a lower Y(II) than wt when reaching the steady state of NPQ. The corresponding parameters in *vccn1* were similar to wt, whereas the double and triple mutant lines displayed intermediate patterns. Based on the determined Y(II) and Y(I) differences (Fig. 3 *insets*), KEA3 and CLCe stimulated electron transport adjusting it to the NPQ on transition from dark to low light.

**Figure 4:**
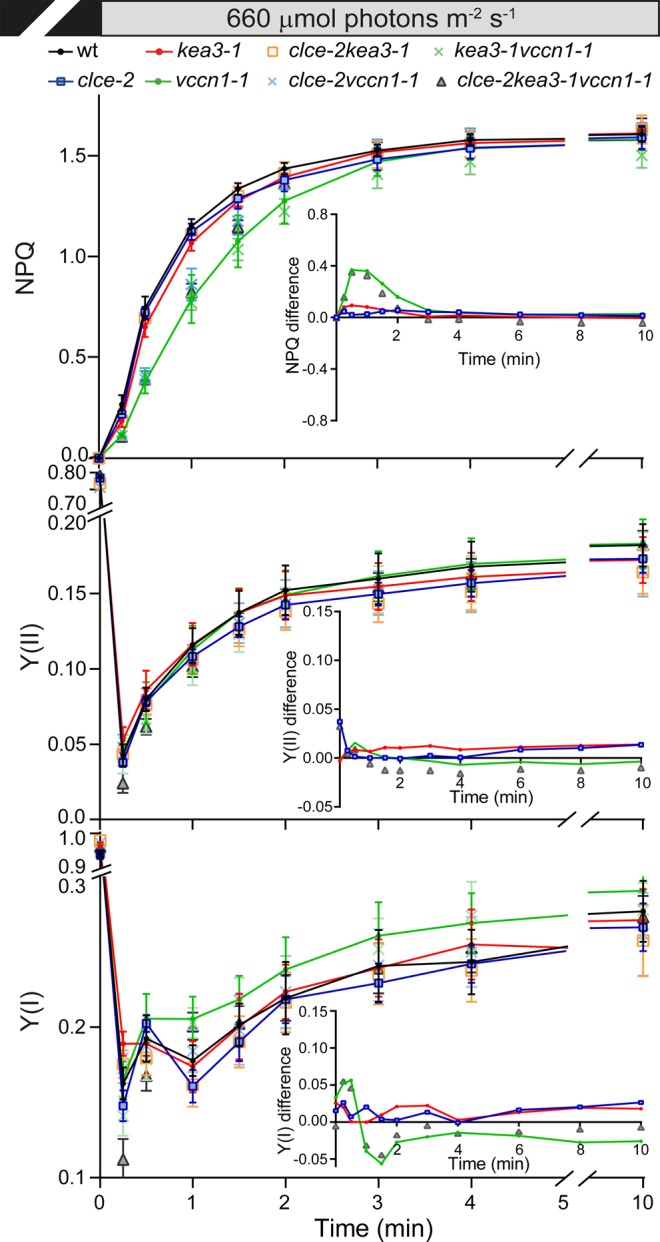
Dynamics of photosynthesis on transition from dark to high light. Wild-type plants (wt) and mutants were grown for 5 weeks and watered with deionized water. Kinetics for induction of non-photochemical quenching (NPQ) were recorded during 10 min of illumination at 660 µmol photons m^−2^ s^−1^. Photosystem II and I quantum yields (Y(II) and Y(I), respectively) were calculated from the same experiment as NPQ. *Insets* show the curve difference of wt minus each mutant. For clarity, only data for single and triple mutants are shown. For NPQ difference plots of double mutants, see Supplementary Fig. 8. Data are the means ± SEM (n = 6 plants). The experiment was repeated twice with similar results.

We have also studied the dynamics of NPQ and photosynthesis on transition from dark to high light. Under these conditions, NPQ was induced more slowly and was indistinguishable among *vccn1* lines, whereas Y(II) and Y(I) were not largely affected (Fig. 4). NPQ and electron transport in *kea3* and *clce* single and double *clcekea3* lines were similar to wt. VCCN1 accelerated the activation of NPQ with a maximum at 0.5 min until 3 min of high light when a steady state was reached, and this effect was not altered by either CLCe or KEA3 (Fig. 4 *inset*, Supplementary Fig. 8). Taken together, these data suggest a sequential and additive action of VCCN1, CLCe and KEA3 in modulating NPQ and electron transport on transition from dark to low light, and a function solely for VCCN1 in modulating these processes on transition from dark to high light.

### Regulation of photosynthesis on transition from low to high light

**Figure 5:**
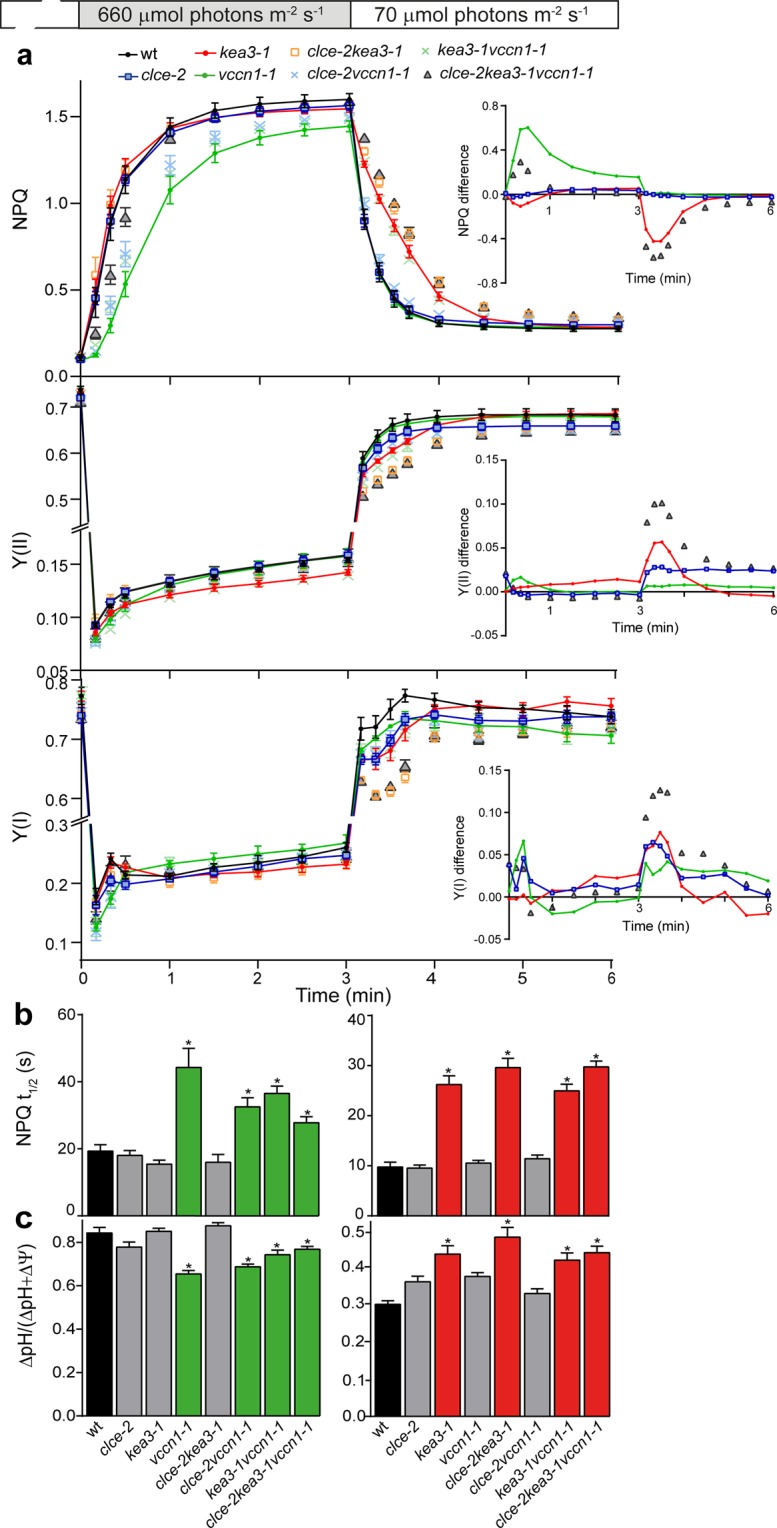
Dynamics of photosynthesis in fluctuating light. Wild-type plants (wt) and mutants grown for 5 weeks and watered with deionized water were illuminated for 10 min with low light (70 µmol photons m^−2^ s^−1^), then for 3 min with high light (660 µmol photons m^−2^ s^−1^) and then again 3 min with low light. (**a**) The plots show NPQ induction, PSII quantum yield Y(II) and PSI quantum yield (Y(I)). *Insets* show the curve difference of wt minus each mutant. For clarity, only data for single and triple mutants are shown. For NPQ difference plots of double mutants, see Supplementary Fig. 8. (**b**) Halftimes for NPQ (NPQ t_1/2_) induction and relaxation were calculated from data shown in (**a**). (**c**) Contribution of the pH gradient (∆pH) to the proton motive force, which is the sum of the electric potential (∆Ψ) and pH gradient (∆pH), was estimated from electrochromic shift measurements at the end of each transition (for examples of recordings, see Supplementary Fig. 9). *Left* and *right panels* in **b** and **c**: low-to-high and high-to-low light transitions, respectively. Data are the means ± SEM (n = 6–8 plants). Asterisks on green and red bars denote a statistically significant difference between wt and *vccn1*, and between wt and *kea3* lines, respectively (one-way ANOVA test, P < 0.05). For size of proton motive force, see Supplementary Fig. 9. The experiment was repeated twice with similar results.

To further investigate the role of CLCe, KEA3 and VCCN1 in photosynthetic regulation, we undertook experiments under fluctuating light (transitions from low intensity to high intensity and back to low intensity). On transition from low to high light, NPQ was induced slower in *vccn1* than in wt and reached a lower steady state (Fig. 5a). NPQ in *clce* and *kea3* largely resembled wt, even though it was induced slightly faster in *kea3*. Double *clcekea3* and triple *clcekea3vccn1* lines displayed intermediate patterns, suggesting additive effects of the mutations. Based on the determined NPQ difference, VCCN1 accelerated NPQ activation with a peak at 0.5 min until 3 min of high light, whereas CLCe and KEA3 only weakly affected the NPQ (Fig. 5a *inset*, Supplementary Fig. 8). The electron transport through photosystems (Y(II) and Y(I)) was similar to wt in all mutant lines except *kea3* where it was lower (Fig. 5a). Y(I) and Y(II) difference revealed that VCCN1 slightly elevated the electron transport within the first 0.5 min corresponding to the peak of NPQ activation, whereas KEA3 and CLCe slightly further increased it during the remaining illumination time (Fig. 5a *insets*).

The calculated halftime of NPQ induction was 1.5-2-fold higher in *vccn1* lines and similar to wt in the other lines (Fig. 5b *left panel*). These data are consistent with a major role of VCCN1 in NPQ dynamics on transition to high light.

In parallel experiments we determined PMF size and partitioning from electrochromic shift (ECS) recordings at the end of the transition from low to high light (Supplementary Fig. 9a *left panel*). The ∆pH significantly decreased in *vccn1* lines, whereas in the other genotypes ∆pH was similar to wt (Fig. 5c *left panel*). PMF size was elevated only in *vccn1* lines (Supplementary Fig. 9a *right panel*). Taken together, these data indicate that on transition from low to high light, VCCN1 functions in building up a significant pH gradient to rapidly activate NPQ without largely affecting the electron transport through photosystems. CLCe and KEA3 do not play a major role in photosynthetic regulation under these conditions and only fine-tune the NPQ produced by VCCN1.

### Regulation of photosynthesis on transition from high to low light

Following transition from high to low light, photosynthesis undergoes a gradual adjustment, where KEA3 was proposed to play an important role^12^. In our fluctuating light conditions, NPQ relaxed more slowly in *kea3* and *kea3vccn1* lines and this effect was slightly enhanced in *clcekea3* double and *clcekea3vccn1* triple lines, while resembled wt in the other lines (Fig. 5a). NPQ differences revealed KEA3 as a major contributor to the relaxation of NPQ in the first min of transition to low light (Fig. 5a *inset*, Supplementary Fig. 8).

Y(II) and Y(I) under the same conditions were lower in *kea3* and *clce* and further decreased in the *clcekea3* double and *clcekea3vccn1* triple lines (Fig. 5a). Based on the Y(II) and Y(I) differences, KEA3 stimulated the electron transport of both photosystems in the first min of transition to low light, whereas CLCe elevated photosystem activity throughout the illumination (Fig. 5a *insets*).

The halftime of NPQ relaxation in *kea3* lines was 3-fold higher than in all other genotypes including wt (Fig. 5b *right panel*), consistent with a role of KEA3 on transition from high to low light. In parallel experiments with ECS recordings at the end of the transition (Supplementary Fig. 9b *left panel*), only the *kea3* lines displayed a significantly higher ∆pH (Fig. 5c *right panel*) while preserving the wt PMF size (Supplementary Fig. 9b *right panel*). Taken together, these data indicate that during the high to low light transition, KEA3 functions in the dissipation of pH gradient leading to NPQ relaxation, while enhancing PSII and PSI efficiency. CLCe also affects PSI and PSII electron transport, and this happens likely by a ∆pH-independent mechanism.

We have also assessed the impact of the mutations on plant growth under fluctuating light conditions (alternating 3 min at 660 µmol photons m^−2^ s^−1^ followed by 3 min at 70 µmol photons m^−2^ s^−1^). The plants of all genotypes had similar rosette size at 4 and 6 weeks (Supplementary Fig. 10) and no significant differences were observed in biomass, Chl content and the F_v_/F_m_ parameter (Supplementary Table 2). These data indicate that the fine adjustments in NPQ and electron transport do not impact plant growth in our design of fluctuating light conditions. Experiments in field-like conditions are required since they may reveal an altered tolerance of the mutants to natural fluctuating light, as it was previously observed for single *vccn1* lines^16^.

### Thylakoid ultrastructure

The thylakoid membrane undergoes dynamic changes in its overall shape depending on the light environment, and this may control important processes in photosynthesis^19^. Recent reports highlighted CLCe and VCCN1 as important players for the thylakoid ultrastructure in the dark- and light-adapted state, respectively^14,17^. Here we studied chloroplast and thylakoid ultrastructure of *clce, kea3* and *vccn1* single and *clcekea3vccn1* triple mutant lines by transmission electron microscopy (TEM). No major changes in the overall structure relative to wt in either dark- or light-adapted plants were observed at either scale of 1 μm or 200 nm (Supplementary Fig. 11). Despite the broad distribution of data obtained from examination of 250–300 grana, the thylakoid stacks (grana) were slightly but significantly reduced in diameter in light-adapted as compared to dark-adapted plants within each genotype (Supplementary Fig. 12). Within the same condition, only *vccn1* had significantly increased granum diameter compared to wt, in accordance with previous observations^17^. Nevertheless, in contrast to the same report, the *vccn1* grana were not curved in either light- or dark state (Supplementary Fig. 11). In another previous study^14^, round-shaped chloroplasts often containing a large thylakoid-free stroma region were observed in dark-adapted *clce* to a higher extent than in wt. Such ultrastructural features could be also observed in the current study, but they were not typical of *clce* since they also appeared in the other genotypes especially in darkness. The reasons for not fully reproducing the previously-described phenotypes of *vccn1* and *clce* are not clear at present. The different watering during plant growth (i.e., deionized water being used in this work and tap water with 1–5 mM Cl^−^ in previous works) and different protocols for fixation and embedding of the samples between this and previous reports may impact the intra-thylakoidal ionic strength, which was demonstrated to result in structural rearrangements in *vccn1*^17^. Taken together, the TEM data indicate wt-like thylakoid ultrastructure in all mutant lines, that could not explain the short-term alterations in NPQ and photosynthesis observed in this study.

## Discussion

K^+^ and Cl^−^ fluxes across the thylakoid membrane have been predicted to function in membrane depolarization and regulation of photosynthesis^24–26^. In this study, we bring evidence that three molecular players in these fluxes, namely CLCe, KEA3 and VCCN1, act independently and by distinct mechanisms in the adjustment of photosynthesis to the light environment.

### Distinct roles of VCCN1 and CLCe in the activation of photoprotection and electron transport

VCCN1 was demonstrated to function as a voltage-dependent Cl^−^ channel in electrophysiological experiments using planar lipid bilayers^17^, and was therefore proposed to mediate Cl^−^ import into the thylakoid lumen. CLCe is believed to transport Cl^−^ as well^14^, but to date, no electrophysiological evidence has been put forward for its substrate or mode of regulation. The mechanism of Cl^−^ transport may be different since CLCe modulates photoprotection and electron transport in different manners and time scales from VCCN1 in the studied conditions. In dark- and growth light-adapted plants, CLCe is likely required for an optimal electron transport activity (Table 1). On transition from dark to low light, VCCN1 accelerates NPQ activation with a maximum at 0.5 min, time when CLCe further stimulates it with a maximum at 1 min of illumination (Fig. 3). In the remaining illumination time, when a steady state in NPQ is reached, CLCe but not VCCN1 stimulates electron transport through photosystems. On transition from dark to high light, VCCN1 alone accelerates NPQ activation with a maximum at 0.5 min until 3 min without largely affecting electron transport (Fig. 4).

VCCN1 and CLCe play different roles also in fluctuating light conditions, since VCCN1 acts in the activation of NPQ on transition to high light, whereas CLCe stimulates the electron transport on transition to low light (Fig. 5a). In support of distinct actions, knocking out both *CLCe* and *VCCN1* genes resulted in a *clce*-like phenotype in electron transport and a *vccn1*-like phenotype in NPQ activation (Table 1, Figs 3–5). The mechanism behind the faster activation of NPQ by VCCN1 involves dissipation of ∆Ψ and increase in ∆pH (Fig. 5b), supporting previous observations^16,17^. The positive effect of CLCe on electron transport takes place by a ∆pH-independent mechanism that could be related to Cl^−^ homeostasis in the chloroplast (Fig. 2, Supplementary Fig. 7 and ref.^14^). Our results support the view of different modes of action for CLCe and VCCN1 in regulation of photosynthesis.

### Role of KEA3 in the downregulation of photoprotection

While VCCN1 and CLCe had no influence on NPQ relaxation, KEA3 accelerated this phase of photoprotection in conditions of decreasing light intensity (Fig. 5a). These are also the conditions at which KEA3 reduced PMF partitioning to ∆pH (Fig. 5b). Our observations support the model proposed by Armbruster and colleagues^13^ where KEA3 is inactive in high light and is rapidly activated upon decreasing light intensity to export H^+^ out of the lumen thus dissipating the pH gradient. KEA3 also favors the relaxation of the NPQ formed on transition from dark to low light, with a maximum at 1 min of illumination (Fig. 3). Thus, KEA3 overlaps in time with the action of CLCe which increases NPQ, resulting in additive effects in the *clcekea3* double and *clcekea3vccn1* triple knockouts. With respect to the electron transport, KEA3 stimulates it in the first 2 min of transition from dark to low light (Fig. 3), whereas CLCe also functions in steady-state growth light conditions (Table 1). The likely mechanisms behind involve the dissipation of ∆pH by KEA3 and regulation of Cl^−^ homeostasis by CLCe, as discussed above. These distinct mechanisms may explain the independent action of KEA3 and CLCe in the regulation of photoprotection and electron transport.

### A model for the action of CLCe, KEA3 and VCCN1 in regulation of photosynthesis

**Figure 6:**
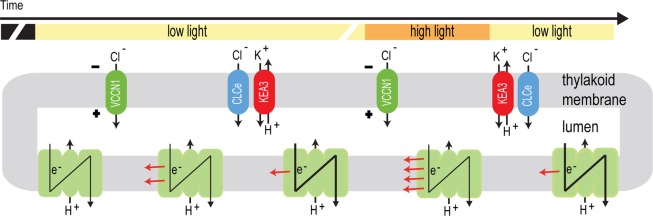
Model for the regulation of photosynthesis by CLCe, KEA3 and VCCN1. The model illustrates the sequence of events during transition from dark to light and in fluctuating light. At the onset of illumination, photosynthetic electron transport generates a transmembrane charge polarity (voltage, −/+) which activates VCCN1. This allows a transient increase in NPQ without affecting electron transport. The function of CLCe resembles that of VCCN1 and partially overlaps in time with KEA3 action, which reduces NPQ to a low steady state while elevating the electron transport. On transition from low to high light, a new voltage is generated which reactivates VCCN1 resulting in increased NPQ. After transition from high to low light, KEA3 reduces NPQ while up regulating the electron transport in a process that also involves CLCe. VCCN1 and KEA3 modulate NPQ by changing the proton motive force partitioning to ΔpH (not illustrated in this model). The model is adapted from Fig. 4c in Herdean *et al*. (2016). A voltage-dependent chloride channel fine-tunes photosynthesis in plants. *Nat. Commun*. 7, 11654, according to article’s Creative Commons license (http://creativecommons.org/licenses/by/4.0/).

On the basis of the kinetic pattern of parameters presented in Figs 3–5, we propose a model for the sequence of events in which CLCe, KEA3 and VCCN1 are involved in the regulation of photosynthesis (Fig. 6). In the first minute upon transition from dark to low light, VCCN1 pumps Cl^−^ into the thylakoid lumen, resulting in a high pH gradient that activates NPQ^17^. A transient maximum is reached, also with the contribution of CLCe, which is closely followed by KEA3 relaxing the NPQ to a low steady state level while elevating the electron transport. The mechanism behind is that Η^+^ in excess are exported by KEA3 in exchange for K^+^ ions, resulting in a reduced pH gradient and relaxation of NPQ^12,13^. On transition from low to high light, VCCN1 elevates the pH gradient and re-establishes a high NPQ while adjusting the electron transport. On transition back to low light, KEA3 exports H^+^ out of the thylakoid lumen and downregulates NPQ, while together with CLCe they reactivate the electron transport. The action of CLCe takes place by a mechanism involving Cl^−^ homeostasis^14^. The presented model shows how three thylakoid channels/transporters work in an independent and sequential manner to adjust the distribution of ions on the two sides of the thylakoid membrane in ways that help plants to fine-tune photosynthesis according to the fluctuating light environment. Future validation of homologues genes found in other photosynthetic and non-photosynthetic organisms^10,27^ will allow us to discover and understand widely conserved adaptive mechanisms to rapid changes in natural habitats.

## Materials and Methods

### Plant material and growth conditions

The *kea3-1* (SAIL_556_E12), *clce-2* (SALK_010237) and *vccn1-1* (SALK_103612) T-DNA insertion mutant lines were obtained from the Arabidopsis Biological Research Center (https://arabidopsis.org/abrc/) and were previously characterized^14,17,18^. Single mutant lines were crossed to generate double and triple mutant lines. For genotyping of the mutants, genomic DNA was isolated from 4-week-old plants and used for PCR. Gene-specific primers are listed in Supplementary Table 3.

*Arabidopsis thaliana* plants of accession Columbia-0 (wt) and mutant lines were grown in a growth chamber (CLF PlantMaster, Plant Climatics, Wertingen, Germany) for 5–8 weeks in soil using a daily cycle of 8 h of light (120 µmol photons m^−2^ s^−1^) at 21 °C and 16 h of dark at 19 °C (unless otherwise stated). The plants were watered once every week with deionized water (resistivity approx. 15 MΩ cm^−1^) for all experiments if not otherwise indicated. In some experiments, the plants were watered once every week with tap water (1–5 mM NaCl) or with deionized water supplemented with 0.5 mM or 5 mM NaCl. In fluctuating light experiments, plants were grown for 6 weeks using alternating high light and low light (3 min at 660 µmol photons m^−2^ s^−1^ followed by 3 min at 70 µmol photons m^−2^ s^−1^) and watered twice a week with deionized water.

### Leaf chlorophyll (Chl) content

Chl was extracted from leaf discs in 96% (*v/v*) ethanol at 65 °C for 10 min and its content was determined by spectrophotometry^28^.

### Measurements of Chl *a* fluorescence and P700 oxidation-reduction kinetics

Fast Chl fluorescence (*OJIP*) kinetics were recorded on 30 min dark-adapted plants by applying a saturating actinic red pulse of 3,600 µmol photons m^−2^ s^−1^ for 1 s using a Handy-PEA (Hansatech, UK) fluorometer. Where indicated, detached leaves were first incubated for 30 min in deionized water or in deionized water supplemented with 150 mM KCl under growth light conditions, then removed from the solution and dark adapted for 15 min before the *OJIP* kinetics were recorded^14^. The maximal quantum yield of PSII photochemistry (F_v_/F_m_), the photosynthetic performance index based on equal absorption (PI_ABS_), and the time to reach F_m_ (t_Fm_) were calculated using Hansatech PEA Plus v1.10 software according to Strasser and colleagues^29^. PI_ABS_ consists of 3 partial performances, namely those related with the concentration of reaction centers, the maximal energy flux reaching PSII centers, and the electron transport at the onset of illumination^30^. Chl fluorescence was also measured on plants during steady-state illumination (4 h in growth light) using a MultispeQ instrument, and the PSII quantum yield (Y(II) was calculated according to Kuhlgert and colleagues^31^, (http://photosynq.org).

Slow kinetics of Chl fluorescence induction and P700 oxidation-reduction were simultaneously recorded with a pulse-amplitude modulated fluorometer DUAL-PAM 100 equipped with DUAL-DB and DUAL-E emitter-detector module (Walz, Effeltrich, Germany). The kinetics were first recorded on attached leaves of 30 min dark-adapted plants using actinic red light of 70 μmol photons m^−2^ s^−1^ (low light) or 660 μmol photons m^−2^ s^−1^ (high light) for 10 min. For experiments in fluctuating light, attached leaves of 30-min dark-adapted plants were first illuminated for 10 min with low light, then with 3 min of high light and then again 3 min of low light. NPQ and Y(II) were calculated based on changes in Chl fluorescence according to Genty and colleagues^32^, whereas Y(I) was calculated from absorbance changes at 830 nm (reflecting the redox state of P700) according to Klughammer and Schreiber^33^. The intensity of the saturating actinic red pulse applied to determine NPQ, Y(II) and Y(I) was 5,000 µmol photons m^−2^ s^−1^ and of 800 ms duration. NPQ, Y(II) and Y(I) differences were calculated as the difference of average value in wt minus average value in each mutant. Calculation of NPQ halftime (t_1/2_) was performed in GraphPad. For this purpose, NPQ induction or relaxation data points were fitted with a single exponential decay and the decay constant (t1) was used to calculate the t_1/2_ as follows: t_1/2_ = t1 * ln(2), where ln(2) is natural logarithm of the number 2.

P700 oxidation-reduction kinetics were recorded based on absorbance changes at 830 nm using DUAL-PAM 100. The P700 signal calculated as the difference in transmittance at 875 nm and 830 nm is displayed as P700 ΔI/I * 10^3^. Before the measurements, the plants were adapted to growth light for at least 1 h. The P700 signal was recorded during exposure of attached leaves to actinic red light of 120 µmol photons m^−2^ (AL, 5 min) and during exposure to far-red of 128 µmol photons m^−2^ (FR, 730 nm, 30 s) after 4 min in darkness. To monitor the P700^+^ reduction kinetics, one saturating actinic red pulse (20,000 µmol photons m^−2^, 200 ms) was applied before AL was switched off and another one at the end of darkness and FR illumination. Where indicated, plants adapted to growth light were also used to record light response curves of Y(I) with 1 min exposure at increasing light intensity via P700^+^-absorbance changes at 830 nm^33^.

### Electrochromic band shift (ECS) measurements

ECS measurements were performed using a Walz Dual PAM-100 equipped with a P515/535 module. First the plants were dark adapted for 30 min and then exposed to fluctuating light as described above. PMF size and partitioning into ∆pH and ∆Ψ were determined from a 60-s dark-interval relaxation kinetics of the ECS signal as described^5^ at the end of the transition at the given light intensity. PMF size was calculated as the difference between the ECS signal in light and the minimum value of the ECS signal after the light was turned off (Supplementary Fig. 9). Calculation of ∆pH and ∆Ψ was performed using the steady-state time point of the ECS signal in darkness. Before each ECS measurement, a saturating 50-μs actinic red flash of 200,000 μmol photons m^−2^ s^−1^ was applied to determine the ECS_ST_; subsequently, the ECS_ST_ amplitude was used to normalize the ECS signal before the calculation of PMF size and partitioning values.

### Transmission electron microscopy (TEM)

Leaves harvested from 7-week-old plants (age when the *OJIP* phenotype was observed in *clce*) that were 16-h dark adapted or 3 h after the onset of illumination were cut into small squares (2 × 2 mm) under dim green light and fixed in Karnovsky’s solution (4% paraformaldehyde, 5% glutaraldehyde, 0.1 M sodium cacodylate buffer, pH 7.3) in vacuum for 4 h in darkness. The fixed leaves were washed three times with 0.1 M sodium cacodylate, pH 7.3. The leaves were post-fixed in 1% osmium tetroxide for 2 h. The samples were washed twice with 0.1 M sodium cacodylate buffer followed by water and thereafter dehydrated with a series of graded acetone. The leaves were infiltrated and embedded in Spurr’s low viscosity resin. Leaf pieces of at least two different plants per treatment were analyzed independently. Ultrathin sections of 80 nm were made by a diamond knife using Leica (EM-UC7) or Reichert-Jung (Ultracut E) ultramicrotomes and mounted on formvar/carbon coated 200 mesh nickel grids. All the samples were contrasted by negative staining with readymade solutions of uranyless and lead citrate (Electron Microscopy Sciences, https://www.emsdiasum.com/) or by lead citrate. Electron micrographs were taken by Philips CM100 or JEOL JEM 1011 microscopes equipped with Morada high resolution digital camera. ImageJ software was used to measure granum diameter on the micrographs. Calculations were done on 250–300 randomly chosen grana originating from 20–25 chloroplasts taken randomly from 10–12 different mesophyll cells per treatment and genotype. The overall structure was investigated for >50 chloroplasts taken randomly from 15–20 mesophyll cells per treatment and genotype.

### Statistical analysis

Statistics were performed in GraphPad software using the Tukey one-way ANOVA test. For all data, P < 0.05 was considered significant.

## Supplementary information

Supplementary information

## Data Availability

The data that support the findings of this study are available from the corresponding author upon request.
