# Supplementary material for: K+ and Cl− channels/transporters independently fine-tune photosynthesis in plants
Source: Sci Rep. 2019 Jun 14;9:8639. doi: 10.1038/s41598-019-44972-z (PMC6570773; doi:10.1038/s41598-019-44972-z)
Supplement: Supplementary file 1 — Supplementary information [file 41598_2019_44972_MOESM1_ESM.pdf]

K^+ and Cl^- channels/transporters independently fine-tune photosynthesis in plants

Emilija Dukic[1,*], Andrei Herdean[1,*], Otilia Cheregi[1], Anurag Sharma[2], Hugues Nziengui[1], Dominika Dmitruk[1,3], Katalin Solymosi[4], Mathias Pribil[2], and Cornelia Spetea[1]

[1]Department of Biological and Environmental Sciences, University of Gothenburg, Box 461 Gothenburg 40530, Sweden;

[2]Copenhagen Plant Science Centre, Department of Plant and Environmental Sciences, University of Copenhagen, Thorvaldsensvej 40, DK-1871 Frederiksberg C, Copenhagen, Denmark;

[3]Department of Botany, Warsaw University of Life Sciences, Nowoursynowska 166, Warsaw 02-787, Poland;

[4]Department of Plant Anatomy, ELTE - Eötvös Loránd University, Pázmány P. s. 1/C, Budapest H-1117, Hungary.

[*]These authors equally contributed to the work.

Correspondence and requests for materials should be addressed to C.S. (email: cornelia.spetea.wiklund@bioenv.gu.se)

Supplementary Information

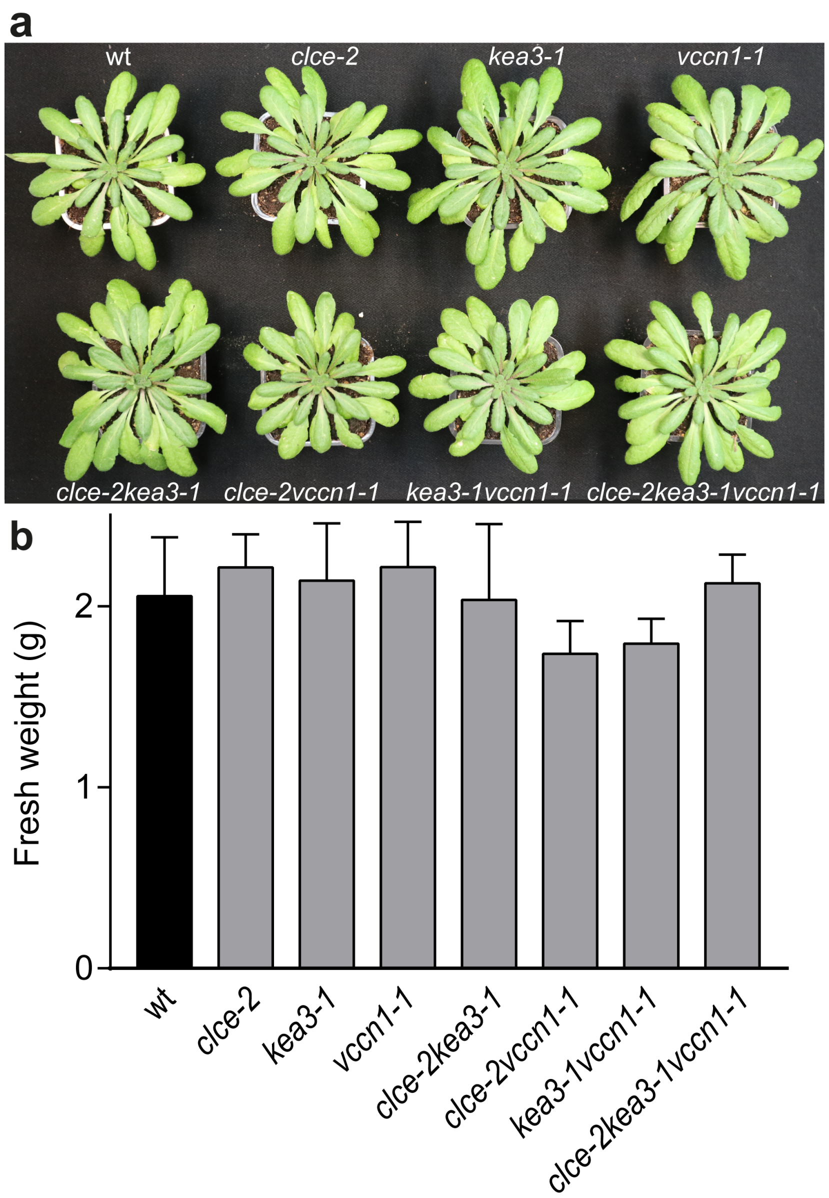

Supplementary Figure 1. Growth phenotype of 8-week-old plants watered with deionized water. (**a**) Representative photos of mutants grown under 8 h/16 h light/dark cycle and a light intensity of 120 µmol photons m^{-2} s^{-1} show no difference in growth with respect to wild-type (wt) plants. (**b**) Shoot biomass of 8-week-old plants was determined as fresh weight. Data are the means ± SEM (n = 8–10 plants). There was no significant difference between wt and mutants (one-way ANOVA test, $P > 0.05$).

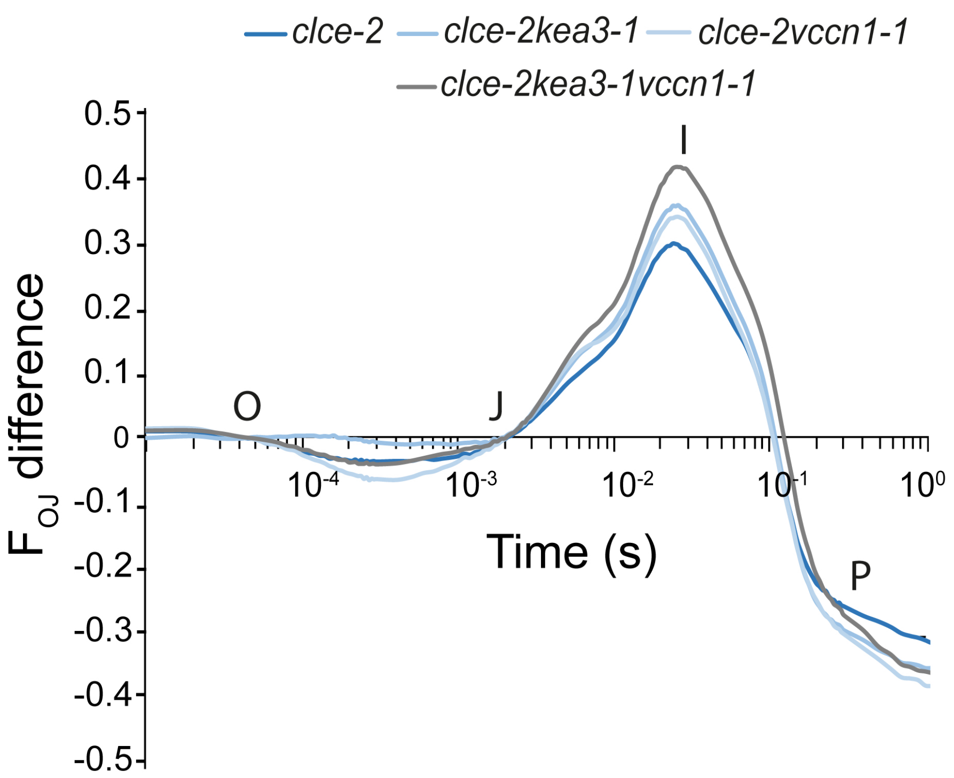

Supplementary Figure 2. The curve difference of double normalized chlorophyll fluorescence data at *O* and *J* between wild-type plants and each of the *clce* lines. The plotted data were obtained from the *OJIP* transients presented in Fig. 2a and are means ± SEM (n = 8–10 plants).

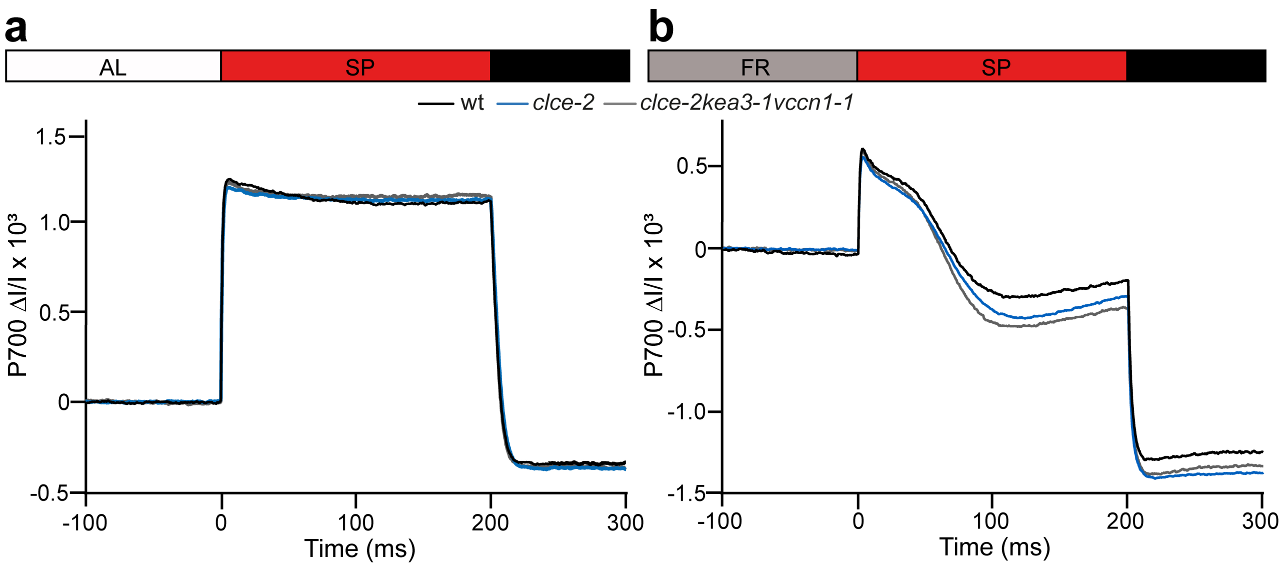

Supplementary Figure 3. P700 oxidation-reduction kinetics in growth light- and dark-adapted plants. Wild-type plants (wt) and mutants were grown for 6 weeks and watered with deionized water. The plants were illuminated with growth light for at least 1 h before recording the P700 signal as difference in transmittance at 875 nm and 830 nm, and displayed as P700 ΔI/I*10^3. (**a**) Attached leaves were exposed to actinic red light (AL) of 120 µmol photons m^{-2} s^{-1} for 5 min followed by a saturating actinic pulse (SP) of 20,000 µmol photons m^{-2} s^{-1} for 200 ms, and 30 s in darkness. The curves were normalized to the steady-state P700 value after the AL illumination. (**b**) Following treatment as in (**a**), the leaves were dark adapted for 4 min, then exposed to far-red light (FR, 730 nm) of 128 µmol photons m^{-2} for 30 s, a saturating actinic pulse (SP) of 20,000 µmol photons m^{-2} s^{-1} for 200 ms, and 30 s in darkness. The curves were normalized to the steady-state P700 value after the FR illumination. The plotted data are means ± SEM (n = 9–17 plants).

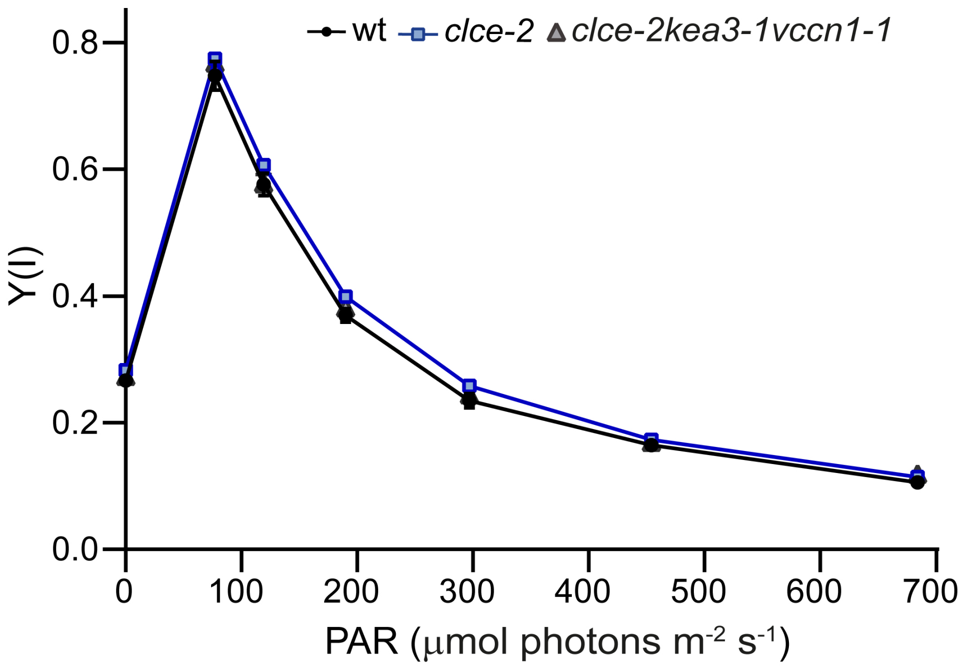

Supplementary Figure 4. Light response curves of photosystem I activity. Wild-type plants (wt) and mutants were grown for 5 weeks and watered with deionized water. The plants were illuminated with growth light for at least 1 h before the response curves of Y(I) were recorded with 1 min exposure at increasing light intensity via $P700^{+}$-absorbance changes at 830 nm. The plotted data are means ± SEM (n = 5-8 plants).

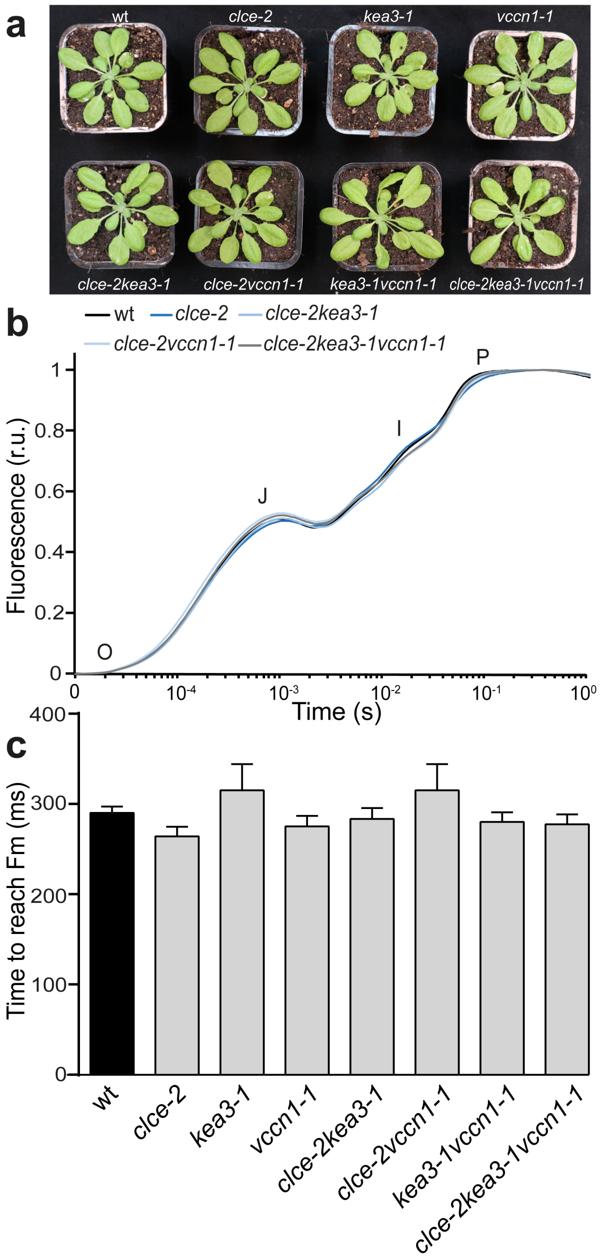

Supplementary Figure 5. Chlorophyll fluorescence phenotype of 6-week-old plants watered with tap water. (**a**) Representative photos of mutants cultivated using 8 h/16 h light/dark cycle and a light intensity of 120 µmol photons m^{-2} s^{-1} show no difference in growth with respect to wild-type (wt) plants. (**b**) *OJIP* transients were recorded on 15-min dark-adapted plants and double normalized to the levels of *O* and *P* steps. (**c**) The parameter corresponding to the time necessary to reach maximal fluorescence intensity F_m was calculated from the *OJIP* transients of (**b**). Data are the means ± SEM (n = 10 plants). There was no significant difference between wt and mutants (one-way ANOVA test, $P > 0.05$).

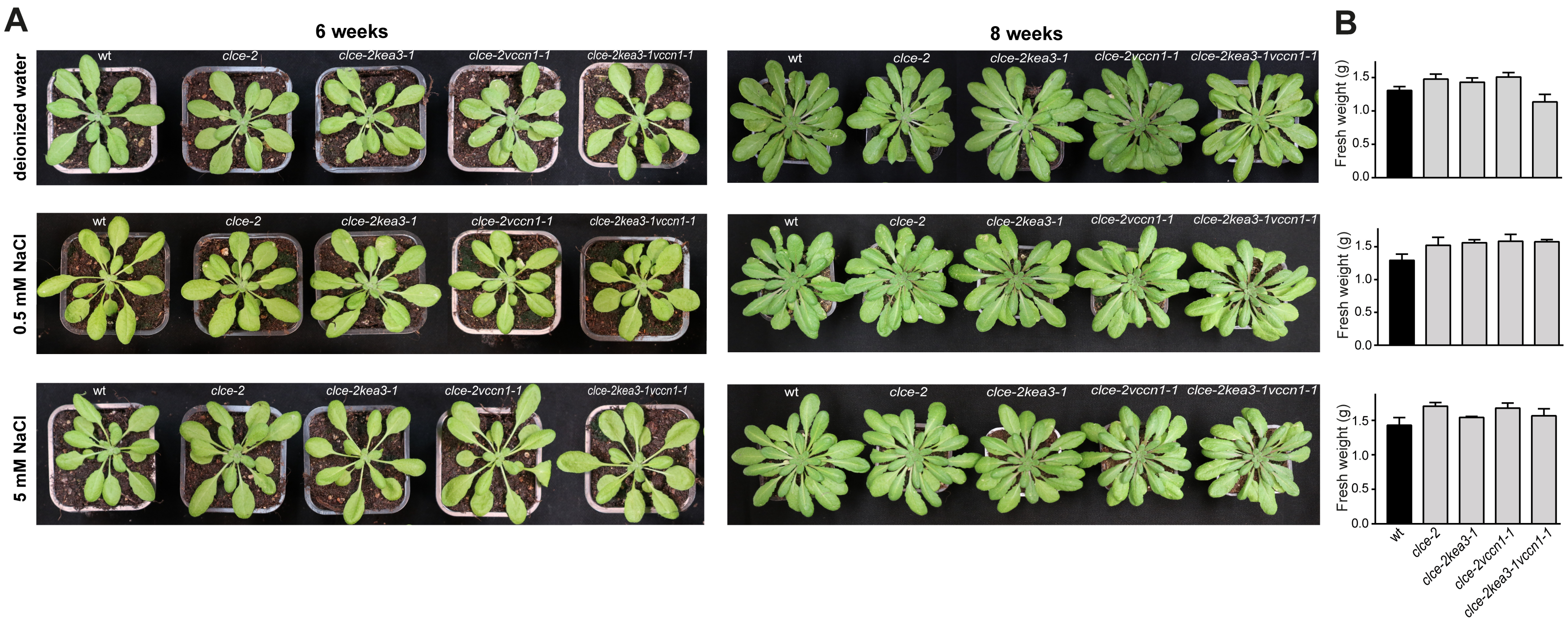

Supplementary Figure 6. The growth phenotype of plants watered with various NaCl concentrations. Representative photos of mutants cultivated using 8 h/16 h light/dark cycle and a light intensity of 120 µmol photons m^{-2} s^{-1} and watered with deionized water or with deionized water supplemented with 0.5 mM or 5 mM NaCl show no difference in growth at 6 weeks and 8 weeks with respect to wild-type (wt) plants. (**b**) Shoot biomass of 8-week-old plants including those shown in (**a**) was determined as fresh weight. Data are the means ± SEM (n = 5 plants). There was no significant difference between wt and mutants (one-way ANOVA test, $P > 0.05$). These plants were used for chlorophyll fluorescence measurements shown in Supplementary Figure 7.

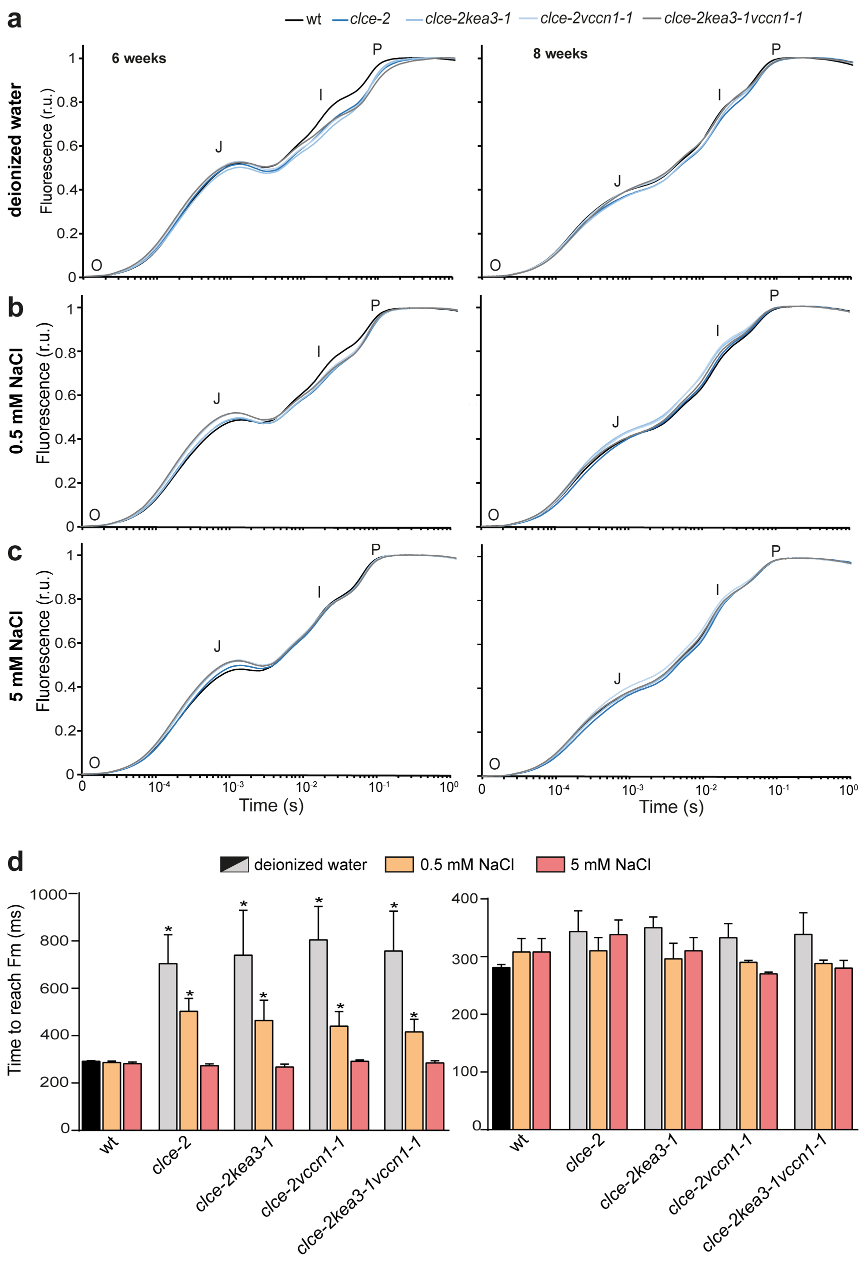

Supplementary Figure 7. Chlorophyll fluorescence phenotype of plants watered with various NaCl concentrations. Wild-type plants (wt) and mutants were grown for 6 and 8 weeks as described in Supplementary Fig. 6, and watered with deionized water (**a**), with deionized water supplemented with 0.5 mM NaCl (**b**) or 5 mM NaCl (**c**). *OJIP* transients were recorded on 15-min dark-adapted plants and double normalized to the levels of *O* and *P* steps. (**d**) The parameter corresponding to the time necessary to reach F_m was calculated from the *OJIP* transients of (**a–c**). Data are the means ± SEM (n = 5 plants). Asterisks denote a statistically significant difference between wt and mutants in the same treatment (one-way ANOVA test, P <0.05).

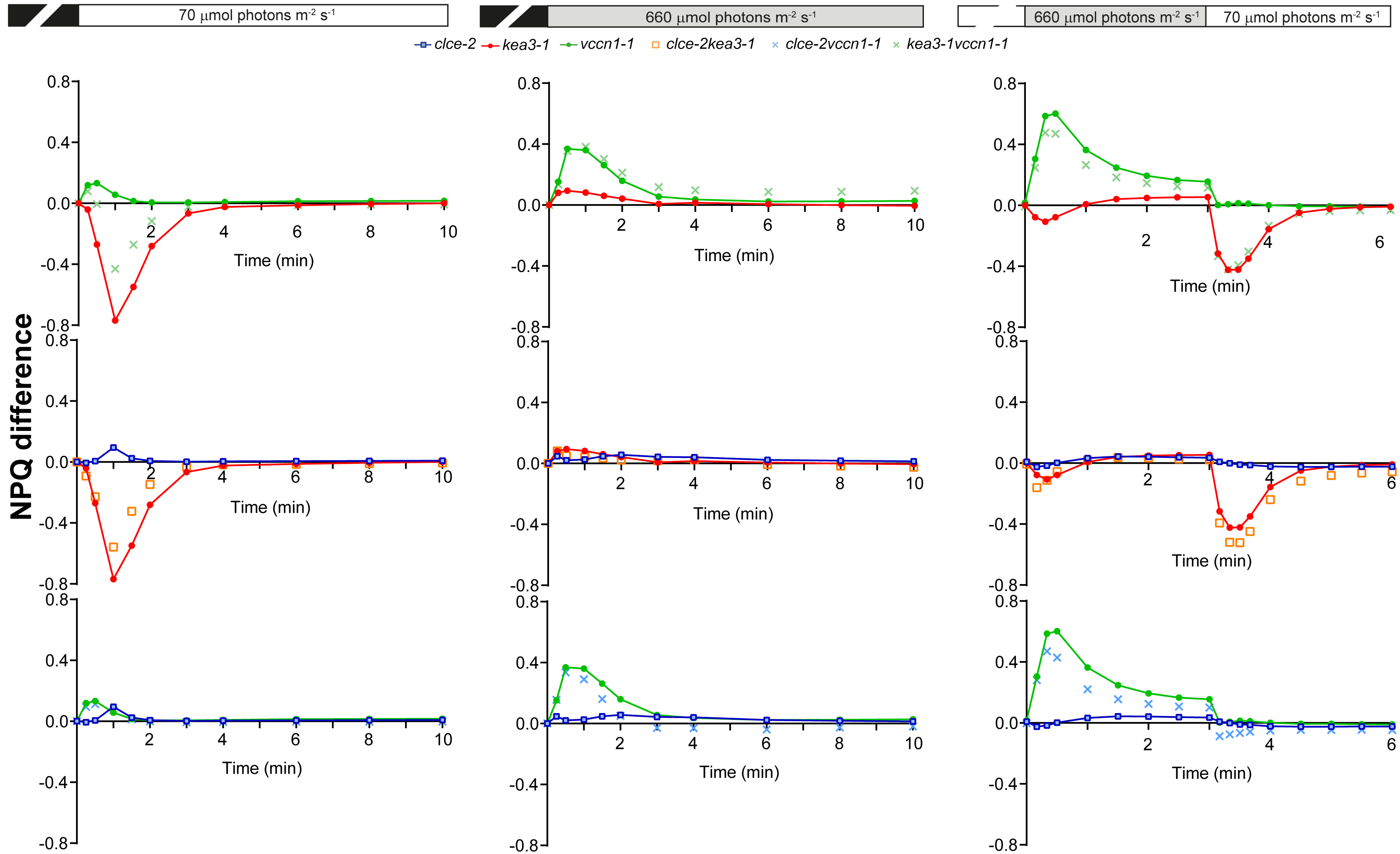

Supplementary Figure 8. NPQ difference between wild-type (wt) plants and single/double mutants. The plotted data were obtained from Figures 3–5.

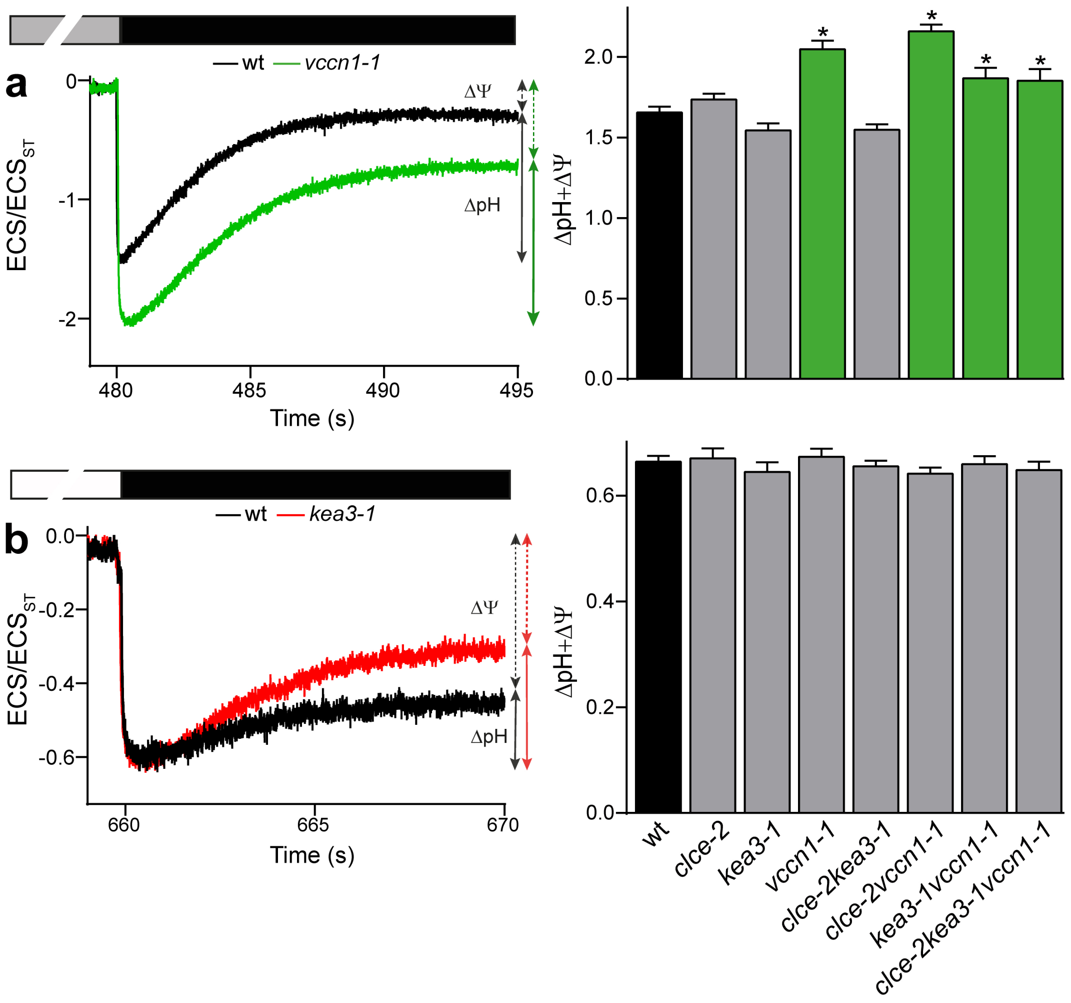

Supplementary Figure 9. Electrochromic shift decay kinetics and size of the proton motive force in fluctuating light. Wild-type plants (wt) and mutants were illuminated for 5 min with low light (70 μmol photons m^{-2} s^{-1}), then for 3 min with high light (660 μmol photons m^{-2} s^{-1}) (transition from low to high light, **a**) and then again for 3 min with low light (transition from high to low light, **b**). Electrochromic shift decay (ECS) in darkness was recorded at the end of each transition. *Left panels*: ECS recordings normalized to ECS induced by a single turnover flash (ECS_{ST}) are shown for wt versus *vccn1* (**a**) and wt versus *kea3* (**b**). The electric potential (ΔΨ) and pH gradient (ΔpH) were estimated as indicated with arrows from individual recordings for each genotype and subsequently averaged to generate the data presented in Figure 5c. *Right panels*: Size of the proton motive force is the sum of ΔΨ and pH. Data are the means ± SEM (n = 6–8 plants). Asterisks on the green bars denote a statistically significant difference between wt and *vccn1* lines (one-way ANOVA test, $P < 0.05$).

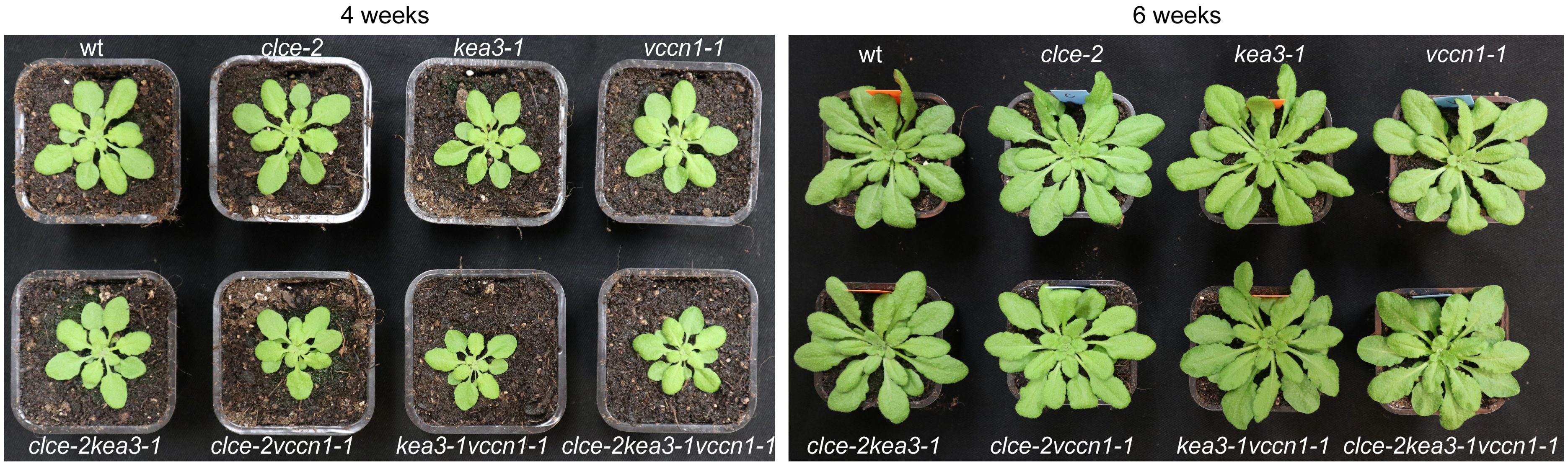

Supplementary Figure 10. Growth phenotype in fluctuating light. Wild-type plants (wt) and mutants were cultivated using a photoperiod of 8 h/16 h light/dark cycle and a light intensity of alternating 70 µmol photons m^{-2} s^{-1} for 3 min and 660 µmol photons m^{-2} s^{-1} for 3 min, and were watered with deionized water. Representative photos of mutants show no difference in growth at 4 weeks and 6 weeks with respect to wt plants.

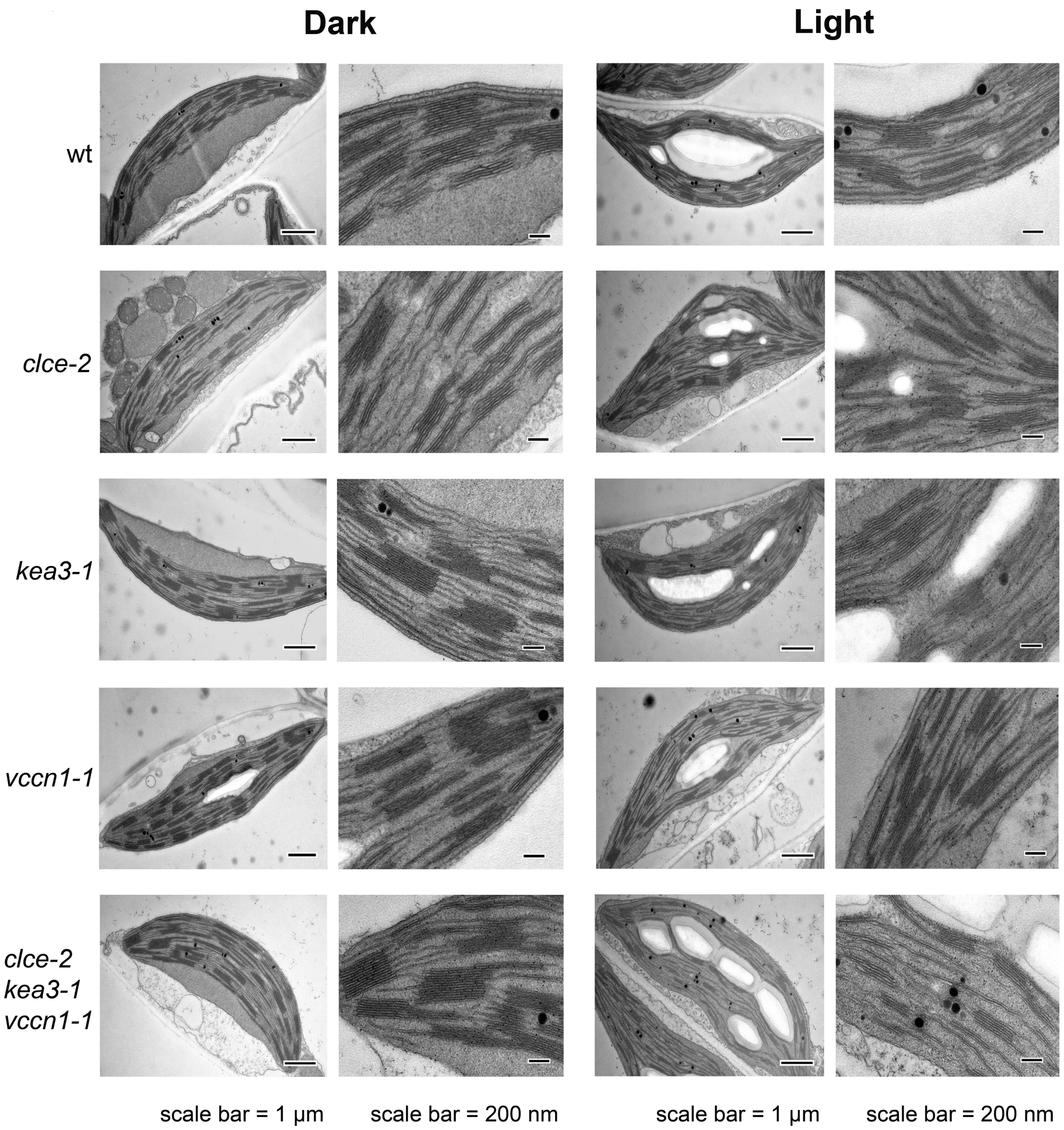

Supplementary Figure 11. Thylakoid ultrastructure. Wild-type plants (wt) and mutants watered with deionized water were grown for 7 weeks (the age when the *OJIP* phenotype was reproduced in *clce* lines in this experiment). Representative transmission electron micrographs are shown for leaf chloroplasts from plants after 16 h of darkness or 3 h after the onset of illumination (120 µmol photons m^{-2} s^{-1}). The mutants display wt-like ultrastructure of thylakoids in both dark- and light-adapted states, based on examination of >50 chloroplasts taken randomly from 15–20 mesophyll cells per treatment and genotype. No major changes in the thylakoid grana were observed at the scale of 200 nm.

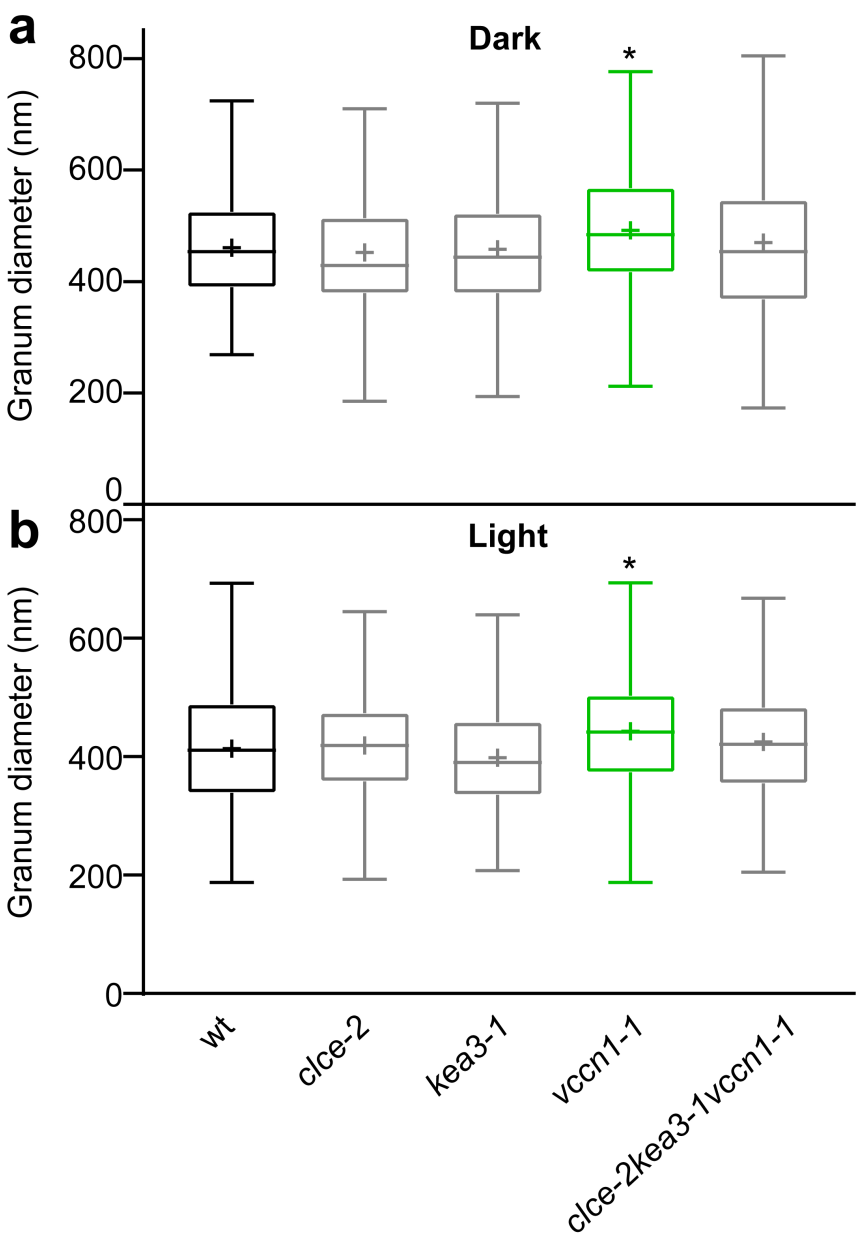

Supplementary Figure 12. Granum diameter. The plants were grown as described in Supplementary Figure 11. Granum diameter was determined from chloroplasts of wild-type (wt) and mutant leaves including those shown in Supplementary Fig. 11, after 16 h of darkness (**a**) or 3 h after the onset of illumination (120 µmol photons m^{-2} s^{-1}, **b**). In total, we measured 250–300 grana originating from 20–25 chloroplasts taken randomly from 10–12 mesophyll cells per treatment and genotype. The 'box plot' (GraphPad) shows data distribution (25, 50 and 75%) ± SD. Significant outliers were not considered in the calculations. Asterisks denote a statistically significant difference in the studied parameter between wt and mutants (one-way ANOVA test, $P < 0.05$).

Supplementary Table 1. Chlorophyll content and fluorescence parameters of 8-week-old plants watered with deionized water. Plants were grown as described in Supplementary Fig. 1. Leaf Chl content and Chl *a/b* ratio were determined spectrophotometrically from leaf discs of 16-h dark-adapted wild-type plants (wt) and mutants following extraction in ethanol. The maximal quantum yield of PSII photochemistry (F_v/F_m), the performance index (PI_{ABS}) and the time to reach F_m (t_{Fm}) were estimated from fast kinetics of Chl fluorescence in 30 min dark-adapted plants. The PSII quantum yield (Y(II)) was determined under steady state growth light conditions (4 h illumination at 120 µmol photons m^{-2} s^{-1}). Data are the means ± SEM (n = 8−12 plants). Asterisks denote a statistically significant difference in the studied parameters between wt and mutants (one-way ANOVA test, $P < 0.05$).

**Parameter**	**wt**	***clce-2***	***kea3-1***	***vccn1-1***	***clce-2kea3-1***	***clce-2vccn1-1***	***kea3-1vccn1-1***	***clce-2kea3-1vccn1-1***
µg Chl cm^{-2}	19.48±0.66	20.48±0.64	19.71±0.62	20.33±0.66	20.50±0.58	18.44±0.72	19.57±0.48	20.71±0.48
Chl a/b	2.30±0.02	2.49±0.02	2.41±0.04	2.42±0.04	2.42±0.036	2.45±0.036	2.40±0.05	2.57±0.05
F_v/F_m	0.824±0.007	0.821±0.005	0.828±0.003	0.826±0.007	0.823±0.002	0.827±0.009	0.824±0.004	0.820±0.005
PI_{ABS}	3.93±0.10	3.60±0.06	4.10±0.18	3.97±0.08	3.97±0.09	3.91±0.19	4.25±0.31	3.30±0.08*
t_{Fm} (ms)	277±8	280±5	269±8	265±8	318±17	353±39	317±43	273±7
Y(II)	0.674±0.015	0.661±0.018	0.679±0.011	0.687±0.011	0.648±0.021	0.677±0.019	0.684±0.017	0.667±0.014

Supplementary Table 2. Biomass, chlorophyll content and fluorescence parameters of plants grown for 6 weeks in fluctuating light. The plants were grown as described in Supplementary Fig. 10. Leaf Chl content and Chl *a/b* ratio were determined spectrophotometrically from leaf discs of 16-h dark-adapted wild-type plants (wt) and mutants following extraction in ethanol. The maximal quantum yield of PSII photochemistry (F_v/F_m) was estimated from fast kinetics of Chl fluorescence in 30 min dark-adapted plants. Data are means ± SEM (n = 6-10 plants). There was no significant difference between wt and mutants (one-way ANOVA test, P > 0.05).

**Parameter**	**wt**	***clce-2***	***kea3-1***	***vccn1-1***	***clce-2kea3-1***	***clce-2vccn1-1***	***kea3-1vccn1-1***	***clce-2kea3-1vccn1-1***
FW (g)	0.84±0.04	0.96±0.04	0.92±0.03	0.98±0.03	0.78±0.07	0.84±0.05	0.83±0.06	0.76±0.03
μg Chl cm^{-2}	15.49±0.31	15.90±0.38	14.80±0.51	16.43±0.66	15.53±0.96	15.01±0.75	16.51±1.24	16.08±0.84
Chl a/b	2.97±0.03	2.93±0.03	2.96±0.05	2.81±0.32	3.03±0.05	3.15±0.06	3.00±0.03	3.13±0.07
F_v/F_m	0.811±0.003	0.802±0.003	0.820±0.001	0.812±0.003	0.816±0.004	0.809±0.002	0.824±0.001	0.809±0.006

Supplementary Table 3. Primers used for genotyping.

Gene	Forward	Reverse
AtVCCN1 (At3g61320)	TGAATCCCCCAATCTATCTTT	AAGAGCAACAGGGAAAGCTG
AtCLCe (At4g35440)	TCCAAGTGTTGAAATTGGAGC	AGGTGTAACAGTCCATGGCAC
AtKEA3 (At4g04850)	AGGTATTTCCTTCCTTGTGCTTACT	AGAATTCCCAAGGTTGCTGA
